# United in Big Data? Exploring scholars’ opinions on academic-industry partnership and the use of corporate data in digital behavioral research

**DOI:** 10.1371/journal.pone.0280542

**Published:** 2023-01-20

**Authors:** Maddalena Favaretto, Eva De Clercq, Arthur Caplan, Bernice Simone Elger

**Affiliations:** 1 Institute for Biomedical Ethics, University of Basel, Basel, Switzerland; 2 Division of Medical Ethics, NYU Grossman School of Medicine, New York, NY, United States of America; University of Connecticut, UNITED STATES

## Abstract

The growing amount of data produced through digital technologies holds great promise for advancing behavioral research. Scholars worldwide now have the chance to access an incredible amount of personal information, thanks to the digital trace users continuously leave behind them. Private corporations play a crucial role in this scenario as the leading collectors of data on users, thus creating new incentives for partnerships between academic institutions and private companies. Due to the concerns that academic-company partnerships might raise and the ethical issues connected with Big Data research, our study explores the challenges and opportunities associated with the academic use of corporate data. We conducted 39 semi-structured interviews with academic scholars (professors, senior researchers, and postdocs) involved in Big Data research in Switzerland and the United States. We also investigated their opinions on using corporate data for scholarly research. Researchers generally showed an interest in using corporate data; however, they coincidentally shared ethical reservations towards this practice, such as threats to research integrity and concerns about a lack of transparency of companies’ practices. Furthermore, participants mentioned issues of scholarly access to corporate data that might both disadvantage the academic research community and create issues of scientific validity. Academic-company partnerships could be a positive development for the advancement of scholarly behavioral research. However, strategies should be implemented to appropriately guide collaborations and appropriate use of corporate data, like implementing updated protocols and tools to govern conflicts of interest and the institution of transparent regulatory bodies to ensure adequate oversight of academic-corporate research collaborations.

## Introduction

Over the last decade, due to the growing sophistication of digital technologies and the extensive use of the internet, the amount of data produced by humanity has grown exponentially. Although there is still debate concerning the quality of the data obtained from the world wide web and other digital sources [1], the digital age, the advent of Big Data, and the Internet of Things (IoT) have all created new opportunities for social and psychological research [2, 3].

While discussing the impact of digitalization in the behavioral sciences, Matthew Salganik writes, “when you think about social research in the digital age, you should not just think *online*, you should think *everywhere”* [4]. Scientists now have the possibility to access a vast pool of personal information about individuals. The digital footprint left by users through the use of multiple platforms and devices—such as social media (Facebook/Twitter/Reddit), streaming platforms (Spotify/Netflix), Google search queries, online purchases, mobile location, smartwatch recordings, and more—creates extensive records of their habits and preferences. These records can be conveniently used to investigate human activity and interaction, predict personality traits or serve as an external validation of classical interview studies in psychology [5, 6]. Even more, the exploitation of aggregated data from social media, GPS, radio frequencies, and consumer data can be utilized to design smart city projects that aim to improve various sectors of urban living, such as education, transportation, pollution control, and energy consumption [7].

In the ecology of Big Data research, private companies play an increasingly important role as the primary entities constantly collecting vast amounts of data. Through the provision of heterogeneous services, most of the time in digital form, corporations can collect a wide variety of data about their users. For instance, membership cards record customer purchases; streaming services register preferences regarding music and movies; smartphones track our location; electronic travel cards record our movements, to give a few examples. As the primary holders and owners of that data, commercial companies are frequently the ones performing research and making advances in Big Data research. Corporations have been using data from users and advanced technological resources to conduct research on their customers to improve their services [8]. For example, OkCupid, a popular dating website, declared testing and working on their users’ data to increase their predictive matching algorithms [9].

Academic-industry collaborations are a well-established reality dating back to the 1930s and have undergone a significant evolution over the past decades [10]. For instance, around the 90s, universities started to be seen increasingly as key economic development actors capable of offering research projects that contribute to industrial innovation in various fields [11]. Partnership with academic institutions represents an attractive opportunity for private companies as it grants them access to scientific and engineering talent in specific domains and cutting-edge research [12]. At the same time, academic institutions and funding agencies recognize private firms as enablers of the collaborative development of capabilities on essential research questions and providers of resources in an environment where funding is limited [13].

This apparently mutually beneficial partnership, however, comes with its challenges. Recognized obstacles to developing long-term, collaborative relationships relate to the discussion of non-disclosure agreements and matters of intellectual property (IP) [12]. In addition, it has been argued that the involvement of the industry’s for-profit aims might impact some of academia’s research objectives, such as basic research in multiple fields [14, 15]. Despite these challenges, the advent of Big Data and the potential it holds towards “solving the world’s most intractable problems (…) from stopping terrorists to ending poverty to saving the planet” [16], plus the wealth of Big Data companies, created new sources and incentives for partnership between the academic and corporate milieus. These incentives were perceived not only in research fields most traditionally linked to corporate collaborations, such as science, engineering, and medicine but also in the humanities and social sciences [13, 17].

Despite these promises, increasingly complex ethical and regulatory dilemmas emerge from the use of Big Data methodologies and corporate data in research. Concerns about consent have been raised when data from companies or digital spaces such as social media is used for research purposes without the user’s explicit consent or acknowledgment [18, 19]. Risk of discrimination and disparate treatment, together with possible harm to vulnerable populations (e.g., children, pregnant people, elders) and ethnic minorities, have been highlighted in the literature regarding corporate practices and research [20]. Moreover, the definition of the human subject in research is becoming blurred as a consequence of Big Data methodologies since the subject of the research is most of the time invisible to the investigator, and the consequent implementation of appropriate regulations to protect research subjects is becoming more challenging [21, 22].

Traditional ethical frameworks adopted by behavioral research are based on two main documents, the Belmont Report [23] and the Declaration of Helsinki [24]. Although primarily developed for medical research, these documents have been used to create ethical guidelines for research practices in other fields, such as psychology and social sciences [4, 25, 26], with scholars constantly striving to adapt clinical research rules to the context of social and behavioral research [27]. At the core of these frameworks are three fundamental *principles*: respect for persons, which is the acknowledgment of participants’ autonomous participation and the need to collect informed consent from study participants; *beneficence*, which is the minimization of harm, either material (physical harm) or immaterial (privacy invasion); *justice*, as in fairness in distribution and dissemination of research outcomes and attention to the selection of research participant.

However, in the context of Big Data research, the interpretation of such principles is inherently challenged. *Respect for persons* is challenged, as mentioned earlier when the research subject is unaware of data collection or does not have control over the analysis of their data [1]. It has become increasingly difficult to appropriately uphold the principle of *beneficence* in Big Data research due to the unpredictability of some of the outcomes of Big Data analysis. This concern, along with the abundance of anonymization issues and privacy infringement in Big Data, might cause unpredicted harm to human subjects [28, 29]. Finally, the discrimination and disparate treatment associated with Big Data methods challenge the principle of *justice* [20]. For this reason, recent research has examined how the values and principles embedded in these documents can guide Big Data research beyond the biomedical field and evaluate where (and why) these principles tend to flounder [4, 25, 26, 30, 31].

Research regulations have struggled to keep up with the ethical challenges that Big Data methods are introducing in research globally. Recent studies have highlighted how there is still uncertainty about appropriately evaluating some of the issues embedded in Big Data research projects. For instance, studies in the United States highlight that Institutional Review Boards (IRB) are currently unequipped to appropriately handle the evaluation of digital research [28, 32, 33] and that there is still little understanding of the unique risks posed by Big Data [26]. For instance, the 2018 revision to the Common Rule, the US policy that regulates research with human subjects in the US, excludes data science research that deals with individuals’ data (such as publicly available or anonymized personal data and social media data) from review. This exclusion, it is argued, might result in more harm than good for research participants [22]. In addition, scholars have also highlighted how the absence of specific guidelines and comprehensive ethical frameworks aggravates uncertainty for ethics committees on what criteria to follow to review and evaluate research projects with Big Data methodologies [34, 35].

In this complicated regulatory context, research done by private organizations does not fall under the definition of human subject research, even if it explores human behavior and cognition through their users’ data. This is because such research focuses on corporate objectives such as increasing and improving user experience rather than finding generalizable knowledge [36]. Corporations can still go through external private independent IRBs to receive an ethical review of their research. However, it is up to the company to choose to use these services rather than a legislative requisite. This differentiation between academic vs. corporate data use and regulation is becoming increasingly concerning for ethicists and data experts, especially as collaborations between private firms and academic research teams are flourishing [26].

These multifaceted ethical and regulatory issues might create a backlash against Big Data research, societal fear about the use of personal data by scholars for research purposes, and reservations toward academic-industry partnerships. For instance, the case of the Facebook Contagion Study [37], which involved the partnership between Facebook and a team of academic researchers from a renowned American University, created controversy and was widely criticized by scholars for ethical violations, including lack of consent and possible harm inflicted to research participants [38]. Nonetheless, as the Big Data era incentivizes partnership and data sharing between companies and academia, it becomes crucial to thoughtfully consider the issues, challenges, and opportunities associated with them to foster beneficial Big Data research.

Our study aims at identifying and exploring some of the challenges and incentives related to partnership and data sharing between private companies and academia in Big Data research. There are numerous different types of academic-industry interaction. This manuscript considers two broad categories of academic-corporate interactions: passive use of corporate data and active collaboration for data collection and analysis. The first is when a team of academic researchers can access company databases or obtain data that the company itself previously collected to perform their research projects. For example, a scholar is given access to mobile-phone network data to conduct dynamic urban research [39]. The second is when an academic team and a company actively collaborate to collect data on a specific sample of users. This can happen when a software developer produces a tool–a device or an app–that the researcher then uses to collect data for an experiment [40].

To investigate these challenges, we have analyzed the opinion and attitudes of academic researchers involved in Big Data research towards collaboration with private companies and the use of corporate data for scholarly research purposes. To this end, we interviewed researchers in the fields of sociology and psychology from universities both in Switzerland and the United States in order to understand: their interest in possible partnerships with corporations and the use of data from companies for their research projects; the challenges they envisage or face when involved in company partnerships; their opinions towards private companies and the research they conduct. The present study directly investigates, through interviews, the views, and experiences of academic researchers regarding the use of Big “corporate” Data and academic-industry partnership. The study also provides suggestions for academic researchers, partners in commercial companies, and regulatory bodies (e.g., ethics committees) on creating a sustainable space for academic-industry interaction.

## Methods

### The NRP75 project–Scope and aims

This study is part of a larger project that explored the regulatory and ethical issues of Big Data research in psychology and sociology. The project is entitled “Regulating Big Data research: A new frontier” and ran between the 1^st^ of February 2017 to the 30^th^ of April 2021 as part of the National Research Programme 75 “Big Data” (NRP 75) funded by the SNSF (Swiss National Science Foundation) [41].

Overall, the study aimed at examining existing regulations and the ethical issues related to Big Data research, addressing the need for harmonization of Big Data research ethical and regulatory practices, and providing concrete recommendations to researchers and ethics committees on how to deal with the emerging challenges posed by Big Data research, specifically in the framework of academic research in psychology and sociology. On the one hand, these two disciplines were chosen because they are at the forefront of using Big Data methodologies in projects involving human research subjects directly and indirectly [22]. On the other, because regulation of academic research in psychology and sociology is being particularly challenged by Big Data research due to the risk of unpredictable harm that it poses for research subjects [28] and because of the challenges that these methods introduce for the concept of human subject research [22]. Particularly in Switzerland, Big Data research is challenging the current regulatory framework for academic research projects (the Human Research Act) [42]. In the US, Institutional Review Boards (IRBs) have faced increased uncertainty regarding how to evaluate digital research projects in these two fields [22]. The study, funded by the Swiss National Science Foundation, was designed to investigate Big Data practices in Switzerland, the home country of the study, where federal institutions are starting to focus on the development of Big Data for research practices. The United States were chosen as a comparative sample because they were identified as a country where Big Data has been a focus of academic research for several years, as evidenced by the numerous federal grants placed for Big Data research [43–45]. In addition, since the overall project aimed to analyze ethical and regulatory practices, the research team selected a country that shared similar ethical research frameworks with Switzerland–the Declaration of Helsinki and the Belmont Report [23, 24].

### Sampling

The study gathered data from 39 semi-structured interviews with 19 American and 20 Swiss researchers (professors, senior researchers, or postdocs. Participants were selected systematically and through snowballing, based on their involvement in Big Data research in psychology and sociology. Inclusion criteria for selection in our study were: 1) academic researchers, from postdoc to professor (Ph.D. students were excluded); 2) involvement in Big Data research; 3) involvement in research in psychology or sociology. Due to the study’s broad aim, collaboration with a company was not considered an inclusion criterion. In addition, no demographic information about the corporate partnership between recruited participants and private firms was systematically collected.

For the purpose of our study, we have defined Big Data as an overarching and inclusive umbrella term that comprises a set of advanced data techniques (e.g., artificial intelligence, neural networks, deep learning, natural language processing) used to analyze large datasets of heterogeneous data to reveal trends and patterns related mainly to human behavior. To identify suitable participants, the research team compiled a list of 17 keywords linked to Big Data, such as Big Data, internet, social media, data linkage, neural networks, etc. (see Table 1). Subsequently, the professional page of professors affiliated with the faculty of sociology and psychology was systematically browsed by the first author for 1) all twelve Swiss universities (ten universities and two federal institutes) and 2) the top ten US universities according to the Times Higher Education University Ranking 2018 (accessed on 13.12.2018). Participants that had these specific keywords appearing on their personal page were selected. Snowballed participants were identified by asking interviewees to suggest the names of up to five possible candidates that would meet the criteria to fit in our study. The snowballed participants were then contacted via email, stating that the correspondent interviewee suggested their names. Since the selection of the sample identified a consistent number of data scientists working on research projects involving data from human subjects, some scholars with a background in data science were included in the sample as their profiles matched the selection criteria set for our study.

**Table 1:** Keywords for participants’ selection.

Keywords for Systematic Web Search
1. Big Data
2. Internet
3. Social Media
4. (Data) Linkage
5. Neural Networks
6. Machine Learning
7. Computational/Computer Based
8. Prediction
9. Data Mining
10. Algorithms
11. Data Analytics
12. Deep Learning
13. Profiling
14 Scoring System
15. (Algorithmic) Modeling
16. Network Analysis
17. Informatics/ Bioinformatics

The research team identified and contacted 194 possible participants– 50 for Switzerland and 144 for the United States. Of those, 39 scholars—20 from Switzerland and 19 for the US—accepted the interview. Table 2 provides a list of the universities included in our sample.

**Table 2:** Number of participants per selected institution.

Switzerland		United States	
Systematically browsed	N. of participants from the institution	Systematically browsed	N. of participants from the institution
University of Basel	5	Harvard University	3
University of Bern	1	Columbia University	1
University of Fribourg	2	Massachusetts Institute of Technology (MIT)	1
University of Geneva	2	Stanford University	2
University of Lausanne	2	Duke University	4
University of Lucerne	1	Yale University	2
University of Neuchatel	0	California Institute of Technology (Caltech)	0
University of St. Gallen	1	University of Pennsylvania (UPenn)	0
Università della Svizzera Italiana	0	Princeton University	0
University of Zürich	2	Cornell University	0
École Polytechnique Fédérale de Lausanne (EPFL)	1	**Through snowballing**	
Eidgenössische Technische Hochschule (ETH) Zürich	2	University of Hawaii	1
**Through snowballing**		University of Southern California	1
Institut de recherche informatique de gestion	1	Georgetown University	1
		Emory University	1
		Vanderbilt University	1
		Northeastern University	1

The 39 interviewees were researchers with a background in sociology (n = 21), psychology (n = 11), and data science (n = 7). Among them, 34 were professors, and five were postdocs at the time of the interview (Table 3).

**Table 3:** Participants.

	Sociology	Psychology	Data Science	Total
**CH Researchers**	9	6	5	20
**US Researchers**	12	5	2	19
**Professors**	20	9	5	34
**Postdocs/Senior researchers**	1	2	2	5

The research team asked for ethics approval from the Ethics Committee northwest/central Switzerland (EKNZ). Since, in Switzerland, interviews with experts (not patients) do not fall under the purview of the Human Research Act, the study was deemed exempt by the ethics commission. Before the beginning of the interview, the interviewer briefly restated the purpose of the overall study, their role in the project, and the confidential nature of the interview to ensure informed consent. In addition, the interviewer allowed time for the participants to ask questions.

### Data collection

The interviews were performed between January 2018 and August 2019 by two research team members. The interviewers were two doctoral students with a background in philosophy and empirical ethics and geography and computer science, respectively. Prior to the start of the interview phase, both interviewers took formal methodological courses as part of their Ph.D. education and received training in interviewing skills.

The interviews were conducted using a semi-structured interview guide designed on a systematic literature review on the topic [20]. The research team designed the interview guide through discussion and consensus regarding relevant ethical and regulatory themes and challenges related to Big Data research. Questions investigated topics such as (1) regulatory practices for Big Data research; (2) opinions and attitudes regarding collaboration with private companies; (3) integration of outsourced data (Social Media data, data from smartphones or sensing devices); (4) opinions regarding data-driven research; (5) ethics of conduct with regards to Big Data studies; and (6) definition and understanding of the word Big Data and attitudes towards Big Data research. Most of the data presented in this manuscript derive from questions related to topics (2) and (3). Other papers have covered different topics [46–48]. The interviews lasted between 35 and 90 minutes. They were tape-recorded and transcribed verbatim. Subsequently, the interviews were transferred into the qualitative software analysis MAXQDA (Version 2018) to support the managing of the dataset and the analytic process [49].

### Data analysis

We applied reflective thematic analysis to analyze the interviews. Thematic analysis is a recognized research approach to data analysis in the context of qualitative empirical methods that aims to arrive at an understanding of a particular phenomenon by investigating the perspective of those involved in it [50]. Thematic analysis is a method for identifying, analyzing, and reporting patterns (themes) that emerge from the data, usually using semi-structured interviews where participants are asked open-ended questions that allow them to share their opinions and perspectives on a topic or phenomenon. In thematic analysis, the importance of a theme is not dependent on quantifiable measures but rather on whether it captures something important concerning the overall research and represents some level of response pattern or meaning within the dataset [51, 52]. We followed Braun and Clarke’s data analysis processes that included several steps: familiarizing with the data; generating the initial codes; searching for themes; reviewing themes; defining and naming the themes; producing the report [51]. The analysis was carried out as follows.

After data familiarization through reading and transcription, the first and second authors initially coded the data from four interviews based on a close line-by-line analysis. They examined the codes to identify potential themes. The two team members subsequently refined their respective categories and provisional themes by discussing them and checking them against the dataset. This was done to reflect on the data and ensure that nothing had been overlooked. Sub-themes were added, and similar ones were combined whenever needed. Finally, clear definitions and names for each theme were generated. Several relevant themes that openly discussed academic and corporate partnership emerged from the interviews, including a) collaboration with companies and opinion on the use of company data; b) integration of data from sensing devices and social media; c) attitudes regarding the conduct of private companies; d) challenges in collaboration with companies; d) regulatory practices for research in private companies.

Due to the relevance of the content that we found within the data regarding academic-corporate collaboration, the research team agreed to report these findings and engage in the description of how academics perceive collaborating with firms and a discussion of these impressions. While discussing corporate partnerships, respondents shared both a) their personal experiences and collaborations with private firms and b) general opinions regarding the challenges and opportunities between academic-corporate partnerships and the use of corporate data. Since a systematic distinction between these two could not be drawn, the research team agreed that all the themes identified would refer to the general opinions of researchers over corporate collaboration. In addition, neither the questions in our semi-structured interview guide nor the participant’s opinion clearly explored the differences/distinctions between active and passive partnerships with corporations. Consequently, the team again agreed to analyze and report the findings as generally referring to corporate partnership, both active and passive. Nevertheless, it is relevant to have both groups represented as this gives an idea of the fundamental challenges that some have encountered and, at the same time, of the (founded/unfounded) fears or hopes of those who do not have any or little experience.

After data analysis, we proceeded with reporting the results of the previous stages. To achieve this, all interviews were analyzed for units of text that related to the themes mentioned above. Such text segments were reread, analyzed, and sorted into sub-codes by the first author. The sub-themes that emerged from the analysis of the text segments included: a) openness of researchers towards the use of corporate data or collaboration with private firms; b) ethical reservations towards corporate research; c) regulatory standards and constraints related to corporate research and partnership with private firms; d) academic vs. corporate research practices.

## Results

Our respondents were participating in research projects that involve the use and analysis of diversified types of data. The table below illustrates the type of data that our respondents were incorporating in their research projects (see Table 4).

**Table 4:** Data used by participants.

Type of data	Sociologists	Psychologists	Data Scientists
Data from companies (anonymized/aggregate purchase data, traffic phone data)	P35CH-S; P38CH-S; P1US-S.		P18US-D; P29CH-D.
Sensing devices and sensor data (smartphone data, GPS, fitness trackers, Wi-Fi interactions)	P28CH-S; P38CH-S; P20US-S; P22US-S.	P22CH-P; P4US-P.	P18US-D.
Social Media data (Twitter, Facebook, GAAB, Telegram, Reddit)	P28CH-S; P3US-S; P12US-S; P20US-S; P21US-S; P22US-S.	P24CH-P.	P29CH-D; P18US-D.
Physiological data (EG, eye tracking)	P22US-S.	P22CH-P.	P8US-D.
Medical data (neuroimaging, blood samples, x-rays, genetic data)	P9US-S; P12US-S; P16US-S.	P1CH-P; P4US-P; P11US-P; P13US-P; P14US-P.	P31CH-D; P32CH-D; P34CH-D.
Administrative data (university and state records, federal records, juridical, tax and census data)	P33CH-S; P39CH-S; P6US-S.	P4US-P.	
Publicly available data (newspaper, books, websites, public documents, data on public figures)	P23CH-S; P30CH-S; P35CH-S; P37CH-S; P1US-S; P2US-S; P3US-S; P6US-S; P19US-S; P20US-S.	P17US-P.	
Interview and survey data	P26CH-S; P28CH-S; P39CH-S; P2US-S.	P24CH-P; P25US-P; P4US-P; P14US-P; P17US-P.	P29CH-D.
Crowdsourcing data (M-Turk, Crowd Flower, Safecast)	P29CH-S; P20US-S.		P27CH-D.
Not specified	P5US-S.		

Key: P = participant+ID number+country (CH = Switzerland; US = United States)+background (P = Psychology; S = Sociology; D = Data Science). Eg. P1CH-P = Participant 1, Switzerland, Psychology.

The analysis of the researchers’ opinions and attitudes towards using company data and collaboration with private firms led to three themes: 1) inclination towards using data from companies or collaborating with them; 2) challenges towards interactions with companies; 3) differences between academic and corporate research. The themes and the respective subthemes are listed in Table 5.

**Table 5:** List of themes and subthemes.

Theme	Subtheme
1. Inclination towards collaborations with companies	
2. Challenges	2.1 Ethical challenges
	• Commercial interests and for-profit motives
	• Transparency in company practices
	• Privacy
	• Consent
	2.2 Methodological challenges
	• Theory driven vs. data driven research
	• Data quality issues
	2.3 Issues of access to corporate data
	• Value of corporate data access
	• Causes of lack of access
3. Status of academic research	3.1 Academic research is slower than/lagging behind corporate research
	3.2 Regulatory inequalities

To illustrate the results, we have reported representative anonymized quotes from the interviews. The findings are reported employing a low level of interpretation, which is customary to thematic analysis approaches [50], to avoid over-interpretation of the data.

### Researchers’ inclination toward company collaboration

As a general trend, participants expressed openness towards collaboration with private companies and the use of data from private firms to perform academic research. When asked if it would be appealing for them to cooperate with companies or to use their customer or behavioral data, some participants highlighted the value of this type of data for their research field. The use of this data could both enhance their current research projects or even create new investigation opportunities (see Table 6, 1. a).

**Table 6:** Relevant quotes over participants’ interest in corporate partnership.

1. Researchers’ inclination towards company collaboration	a) This data [commercial data] is a gold mine because you get purchase data, scanning data, you get travel data from these mobile phones, and so on. So, this is extremely revealing. (P38, Sociologist, CH)
	b) Well, I think. . .this is going to sound erratically to you, but I actually think that [collaboration between companies and academia] it’s essential. And the reason I say that is because, at the end of the day, what we do with the discoveries that we make in the academy doesn’t get to work with the patients in new products unless we collaborate with companies. (…) It’s really clear that companies have a role to play in the ecology of delivering products to people. And, you know, universities they don’t make things… (P14, Psychologist, US)
	c) So, another question I think for research will be interesting, and that’s going to be changing within the next couple of years, is that we buy more data from commercial providers. In addition to databases of… I don’t know newspapers, for example (…). We may also start buying data from companies who have, I don’t know, forty-thousand Swiss consumer interests in it and. . . just like commercial enterprises buy data. (P23, sociologist, CH)

In this context, a couple of interviewees emphasized Big Data’s impact on scholarly research and the state of academic-industry collaboration. Cooperation between companies and researchers was seen as essential for academic research to have an impact on society since corporations are the entities that have the resources, both financial and technical, to invest in developing and delivering beneficial products and technologies for the public. In addition, it was envisaged that universities would be more inclined to obtain data from commercial providers to conduct Big Data research in the future. This circumstance would see an increment in the use of data acquired from external sources rather than research groups performing data collection themselves (see Table 6, 1. b, c).

### Challenges towards interaction with companies

While expressing their opinion toward academic-industry interaction, many participants pointed out some challenges that might hinder the relationship between academia and companies. We subdivided such concerns into three categories: 1) ethical reservations, 2) methodological concerns and 3) access issues.

#### Ethical reservations

Although they recognized the value of corporate data for research purposes, a consistent number of participants reported their uneasiness regarding collaborations with private firms or expressed reservations about the academic use of commercial data due to concerns regarding the ethical challenges such interactions might bring.

A frequent concern shared by our participants was related to the commercial interests of private companies. In this context, some researchers, even though companies typically spend 2–23% of revenue on research and development [53], underlined an ethical tension between the purpose and values that characterize scholarly investigation (advancement of knowledge) as opposed to the interests that move corporate research (making a profit) (see Table 7, 1. a).

**Table 7:** Relevant quotes over perceived challenges for corporate partnership.

1. Ethical Reservations	a) Because mostly we [academics] are doing research, and they [companies] are doing business, right? They want to earn more money. We’re not interested. . .well, that is not our concern, right? We want to do research, right? (P29, Data Scientist, CH)
	b) I think that the goals of certain commercial research very often are to accumulate data that can be used for profit ends. Which, again is not inherently a bad thing. But I think that one of the things that can happen is that the infallibility of data for profit sharing and co-commercialization can create certain ambiguities—if not tensions—with regard to the values under which data are collected and utilized. So I think that there can be an issue there, you know? Data for sale and multiple commercial uses that have profit motives can be problematic. (P11, Psychologist, US)
	c) Now, the ethical issue behind it is, of course, companies conduct those experiments all the time. It’s only that researchers are supposed to have their hands bound more than companies do. So it’s not so much necessarily a specific experiment that is a problem. More the ethical concerns: who is conducting the experiment and for what purpose. (P2, Psychologist, US)
	D) So I think that. . . there need to be guidelines in place so that the company doesn’t exploit the researcher. So I think sometimes researchers are so desperate to get access to information and data that we agree to work for free. And that’s not fair, I mean, that’s a different kind of ethical problem right? So companies should have to pay for our time (laughs). (P22, Sociologist, US)
	e) I think, as part of the education for society, it would be important to have more transparency about how this data from all those companies, how it is used in. . .people have to have some choice on…well if they’re going be using this (…) So, the transparency part is important, I would not limit research being done on that kind of data because the more we know, the more we can help improve the system, right? I mean, always in within…a humanist way, how to improve it for a better society. (P5, Psychologist, US)
	f) During the two weeks when we collected the data via the phone, people actually wore a Fitbit type of device. At the time, it was a company called X (*name anonymized*). It’s very interesting, by the way…the company went under, and one issue in this world of using technology to collect data is that this happens to many companies. It’s remarkable how many companies will show how they’re going to be fantastic, and they’ll lure you in, and you use their product only to find a year later they’d gone under, or they were bought by someone, and they changed it. (P4, Psychologist, US)
	g) I mean, so you have to a certain extent to trust the commercial. But you really have to be careful about who it is, and the big companies are the worse by far. And the startup normally gets bought up, and then they turn into the same thing. So you know, I think it’s really kind of a dangerous time right now, I mean, we do need to have regulation of some kind. (P8, Data Scientist, US)
	h) If the data is public and the participants knew that it was going to be public, then it’s fine. But if it’s something that, you know, first of all, you have to purchase and a company collected that data without the participants consciously knowing that the data’s being collected, I don’t want to participate in that. (P12, Sociologist, US)
	i) I do know that one of the big problems with these kinds of data is the potential for re-identification. The data themselves might not be formally linked to any individuals or households, but particularly if data sources are brought together, it can be straightforward to figure out who’s who. (P16, Sociologist, US)
2. Methodological Challenges	a) Well, the problem with commercial data is that is not intended to do research with most of the times. (…) I think this is a big problem in a way, because when you have these big data or these data stuff, you’re not completely free in theorizing and making your hypothesis and so on. (…) But this is not sound psychological scientific endeavor in a way that, yeah, you need first a theory, the hypothesis, and then you look at the data. (P9, Psychologist, CH)
	b) I mean. . .it obviously it depends on how the data are acquired. Whether it corresponds to a population. And how much we could control it because it’s always the question of quality control. So, if you want to publish with it we need to be able to make sure how the data exactly are acquired, how the people are sampled, and unless that is known, it is relatively difficult to do anything with the data because sometimes is quite hard to interpret. (P32, Data Scientist, CH)
	c) The quality of data that advertisers and marketing is willing to rely on versus the quality of data that I would want for academic research is very, very different. So I would maybe be able to study what sorts of things they can do with the data, but I would not be able to know how accurate they are. I would not be able to, based on the data quality, which will be very loose. Because there’ll be tons of inaccuracies. (P18, Psychologist, US)
3. Issues of access to corporate data	a) Google or with Facebook or with other companies, that they are internally using all this data, and they’re making advances, and they’re sort of withholding the data from the greater researcher community. Then yes, that case is problematic. (P12, Sociologist, US)
	b) I think that, in general, I’m interested in doing much more skeptical and critical research, and if I get data from a company under certain agreements, I would be concerned (…) that I wouldn’t be able to use that to kind of critically analyze the data and talk about its limitations. And certainly, if I were within a company doing that research, I would have that concern. (P18, Data Scientist, CH)
	c) So, in that case, it’s very typical that the provider of the data only allows you to work with the data in a secured place within the company. So then again of course, the problem emerges of how can we verify that these results hold. Because you wouldn’t get access to reproduce it or whatever. (P30, Sociologist, CH)
	d) Now, what happens is that a lot of companies they realize that data is value, but they don’t always understand what kind of value it is. And then they don’t want to share it because they don’t know. . . The good thing is that, compared to money, when you share the data, you actually increase the value on both sides. When you give money to somebody, you lose money, but if you share data with somebody, I think both parties are better off. But a lot of companies still deal with data like with money and they don’t like to share it because they believe they would lose some. . .some power. (P38, sociologist, CH)
	e) Because they [the company the researcher was collaborating with] were not really too much excited about providing any data. Because they wanted to protect their customers and maybe because they also wanted to protect their business model, right? They’re not interested in sharing how much sales in which regions… (P29, Data Scientist, CH)
	f) I see that the major difference in the sense that companies like Facebook, Google, Amazon, Uber, whatever, you pick the ones you like, are sitting on like massive amounts of data that would be amazing for social scientific projects. And they are hiring lots of PhDs to work in their data science teams, except that PhDs don’t usually have the right to publish or, when they publish, they can only publish things that are not detrimental to the company’s image and brand, right? Obviously like kind of sampling in a bit of weird way because they can’t say anything negative about the company…these companies don’t usually like sharing their data with academics, with a couple of exceptions like Facebook and the emotional contagion experiment but then. . .there was this big backlash, so you know, now they don’t want to do it anymore really. (P2, Sociologist, US)

Commercial interests and for-profit motives were often mentioned as factors that might decrease research integrity, as they might create ethical tensions and ambiguities within academic research endeavors, especially concerning the values that drive data collection and use (see Table 7, 1. b). In addition, assessing the purpose and intentions behind a research project and the applications of research outcomes were considered determining factors to be evaluated in relation to corporate-research partnerships. In this regard, companies’ involvement and commercial motives might raise conflicts of interest between the investigator and the company. Hence, according to the participants, it is not necessarily the design (e.g., analysis of personal data or prediction of sensitive characteristics) of a project that makes it ethically problematic but the entity conducting it, its motives, and its purposes (see Table 7, 1. c). Furthermore, in the context of economic interests, some researchers were concerned about academic researchers being exploited by companies for their gain. For instance, when academic researchers are not appropriately compensated for their work on corporate data (see Table 7, 1. d).

A few of our participants were also concerned that many private firms are conducting research without sufficient transparency regarding their purposes and practices, such as data collection and the use of personal data. On this, a researcher highlighted how scholars in the social sciences are concerned mainly by the fact that “privately held companies are collecting vast amounts of social data in ways that are not transparent” (P19, Sociologist, US). For some of our participants, transparency thus emerged as a crucial research standard to be promoted in academic-industry partnerships. According to a participant, research with corporate data is essential to advance knowledge and improve society. However, education and transparency regarding corporate practices should be enhanced in order to benefit all members of society (see Table 7, 1. e).

Two researchers were concerned about startup companies being acquired by more prominent firms as this might create issues of policy change, trustworthiness, and transparency regarding how the collected data will be handled or used after the acquisition. For example, one of our participants, who had first-hand experience with data collection through a startup, pointed out that data collection from a device could be transferred from one company to another without having guarantees that the new company or institution would respect the previous agreement signed by the user (see Table 7, 1. f). Another participant similarly shared that big corporations are usually associated with lower ethical standards and a lack of transparency regarding their data practices. Scholars should thus be careful when partnering with small startups that big firms could, later on, buy (see Table 7, 1. g).

Finally, consent and privacy issues were sometimes perceived as a deterrent to the use of corporate data. On the one hand, participants shared their uncomfortableness about using data without the subject’s explicit consent or awareness. On the other, they highlighted issues of re-identification and anonymity that could emerge from the analysis of certain types of corporate data, making it problematic to analyze corporate datasets safely (see Table 7, 1. h, i).

#### Methodological challenges

On top of these ethical reservations, our participants also highlighted some issues related to the value of corporate data for academic research. For example, several researchers pointed out that data collected by companies might not be suitable for academic research practices in psychology and sociology as they are mainly theory-driven fields. In contrast, big amounts of aggregated data collected by companies are mostly suited for post-hoc analysis (see Table 7, 2. a).

A few participants also had reservations regarding the quality of the data collected by companies. A crucial concern in this context was that data from companies is difficult to use and interpret, as it often lacks some essential information to be properly used in the academic environment. For instance, a participant underlined how issues for academic publishing might emerge when scholars use data from companies as they would have minimal control or knowledge over data collection practices (see Table 7, 2. b).

Additionally, according to a couple of researchers, data from companies is qualitatively not accurate enough to be used in academic research as it is usually full of inaccuracies and thus not trustworthy for methodologically sound research practices (see Table 7, 2. c).

#### Issues of access to corporate data

Another challenge many of our respondents emphasized was that companies do not seem interested in collaborating with academic researchers. According to our participants, companies do not allow access to their databases or share data with university-based scholars (see Table 7, 3. a). Furthermore, some of our participants declared that even if they got access to corporate data, it would not carry an added value to their research. They would, in fact, not be allowed to publish their results or to perform the type of research they are interested in freely. Participant 9 (Psychologist, CH), for instance, reported: “the companies where I get the data from, they say: << you can describe the method you developed, but not the results >>. So I always have big problems with publishing my studies”. Another researcher noted that companies might not allow them to perform critical research about the limitations of corporate data or methods by imposing data agreements that would prevent them from pursuing their desired line of research (see Table 7, 3. b).

Lack of access to companies’ original data repositories, or restrictions regarding the publication of results, were connected by two of our participants to issues of reproducibility. In their opinion, both research performed internally by the company and conducted by an external scholar cannot be verified by other researchers as they would not have access to the same resources (see Table 7, 3. c).

Some of our respondents provided reasons for this reluctance to share data with the academic milieu. One participant claimed that companies tend to mistakenly treat data like money, assuming that sharing their data will result in a loss for the firm. In the participant’s view, data sharing would actually increase the value of the data and benefit both the company and its collaboration partners (see Table 7, 3. d). A few participants associated this reluctance with protecting customers’ privacy and preserving corporate business models (see Table 7, 3. e). Finally, a couple of researchers linked this issue to a reputational concern. According to them, companies might prevent scholars from publishing their results or conducting critical research with their data because they fear such research might tarnish their reputation. Academic researchers having access to company resources could willingly or accidentally expose some of the company’s practice that might be considered unethical or attract public and academic scrutiny. This happened in the case of the Emotional Contagion Experiment, where the partnership with an academic institution resulted in a huge societal backlash for Facebook (see Table 7, 3. f).

Finally, challenges of data access were also associated with a lack of skills. For instance, a couple of researchers pointed out that they lack the appropriate research skills to properly analyze and benefit from the large datasets companies offer. A Swiss sociologist, P24, shared: “I would not touch Twitter but I would collaborate if somebody then has the skills, because otherwise, I would have to acquire all these skills”.

### Are corporations and academia on the same page?

While voicing their opinions on private firms and Big Data, some participants also discussed the current state of Big Data scholarly research compared to the condition of companies and corporate investigations. For example, a couple of participants claimed that companies have been dealing with Big Data long before academic scholars; therefore, they might be more prepared to deal with both the challenges and the potential that Big Data has to offer (see Table 8, 1. a). In this context, while admitting that scholarly research in Big Data is lagging behind compared to corporations, a participant suggested that this would be the right time to reflect on how academic institutions should move forward with corporate Big Data: to what ethical standards academics should comply, what type of data should be investigated and invested in, what type of collaborations they should entertain with private corporations (see Table 8, 1. b).

**Table 8:** Relevant quotes over differences between corporate and academic research.

1. Status of academic research vs corporate research	a) Three, four, say five years ago in our society, very few people were talking about Big Data. Most people didn’t even know it existed. This has been going on for years, probably several decades. So companies, certain kind of companies, and services have been doing this before scientists were actually looking into the implications and how to do it right. (P35, Sociologist, CH)
	b) What do we do with the fact that there’s data sets that are being used to make predictions about what we are going to buy at the supermarket and what I really need as a consumer? (…) And the question is, what do we want? Do we want, and I don’t have an answer to that, but do we want only publicly funded, very thorough panel studies of retrospective ideas of what I bought yesterday? Or do we want that kind of data and how can we combine even the retrospective and the actual tracked data of consumers? I think that’s. . .those are interesting questions that we have to answer. Now, I’m not saying that we should become, you know, yet another tool of marketing. But I think it’s an interesting challenge to think about what data sources are available and which ones do we want to use, and which ones do we want to have access to. (P23, Sociologist, CH)
	c) The good thing is that the clock of a company is not the same as the academic clock (…) So the first version of the paper, they [the company] were really afraid of the sensitivity of the results. But six months or eight months after, it did not matter to them anymore, and we could proceed with the publication. So this is something I’ve seen several times, and that helps also to publish even if it is at some point perceived as sensitive. So it was aging quickly for the business, but it was still relevant for research. (P38 Sociologist, CH)
	d) We don’t want to go into troubles because we don’t have time, that’s one thing, and the other thing, we don’t have the legal support that basically big companies have. So…Facebook or Google or whatever, they will continue doing it because if this happens, they can afford it. We cannot afford it. Right? We don’t have time, simply we want to avoid that, right? That’s the point, right? That makes it a bit complicated. So the assumption is simply that we as research institutions would have a kind of lawyer next to us who would simply…like each time we face a problem, we get in touch with the person, which is not really the case. (P29, Data Scientist, CH)
2. Regulatory Inequalities	a) And this is probably because the regulations are much more restrictive for scientists in institutions than for companies because companies don’t undergo a cantonal ethics approval and they have a business secrecy, and so there are a lot of things going on that couldn’t be studied by scientists. (P35, Social Scientist, CH)
	b) I think that the biggest problems are not universities but private companies. Private companies are collecting inordinate amounts of data, some of them have almost data monopolies, and we don’t have access to that data. And we saw what happened, we’ve only seen the tip of the iceberg of what is happening with Facebook. And nobody has the power to regulate Facebook, the markets are not going to regulate Facebook, consumers are not going to regulate Facebook. (…) Universities are nonprofit organizations and they have. . .they’re subject of scrutiny in more ways than private companies are. (P1, Sociologist, US)
	c) I do want fairness, I do want rules and all, there should be some ways to have rules for everybody. And if you can’t enforce rules for everybody, then quite frankly, there should be rules for nobody. I can take a very libertarian point of view. (P13, Psychologist, US)
	d) Big companies, even Facebook, they have real reputational risk at stake. I think that the issues that the big companies, the big holders like Facebook, like Twitter, like, Reddit, like all these things… you know, I think that what’s ultimately going to bring them in line is just the concern that people aren’t going to be happy with what they do, and that’ll be a big issue. And you notice that that’s actually not a legal thing. That’s just going to be the major driver as opposed to any sort of legal solution. (P14, Psychologist, US)

A Swiss respondent highlighted the differences in research standards between companies and academic researchers by mentioning the concept of the “research clock”. In their opinion, scholars have the possibility to conduct valuable research on datasets that are considered outdated by companies. The researcher illustrated this at the hand of the time lapse between data collection and the review process of academic journals. By the time one of their manuscripts went through the review process of an academic journal, the research team was allowed by the company to disclose information that was considered sensitive at an earlier time. This allowed the scholars to successfully publish their research (see Table 8, 1. c).

A few participants also complained that academic researchers do not have the same support system that companies possess. In their opinion, companies that deal with Big Data operate together with units with diversified expertise–computer science, data security, law–that assist their researchers with all facets of Big Data research, such as compliance with regulatory standards, methodological and infrastructural support, and others. For instance, while discussing some of the regulatory issues they faced with their research project, one scholar shared their frustration of not having adequate support and, therefore, always being at risk of doing something wrong (see Table 8, 1. d).

Numerous researchers discussed the difference in regulation between corporate research and academic research. In particular, researchers from the US and Switzerland saw it as problematic that companies do not have to obtain ethics approval as opposed to scholarly researchers. In some cases, the circumstance of being subject to more restrictive regulations was felt by some scholars as a frustrating double standard where regulations are lacking to govern big corporations. At the same time universities are subject to excessive scrutiny (see Table 8, 2. a, b). In this context, while discussing the regulatory constraints of academic Big Data research, one of our participants complained that their research was overregulated out of excessive cautionary attitudes and suggested that universities and companies abide by the same rules (see Table 8, 2. c). On the other hand, according to one of our researchers, reputation might become one of the driving regulative forces toward research integrity in corporate research more than legislation and regulatory bodies. Should customers and users be discontent about how their data is handled, companies will have to face possible adverse reactions (see Table 8, 2. d).

## Discussion

Big Data methods and digitalization are incentivizing interactions between private companies and academia. This study contributes an analysis of the incentives and barriers to creating sustainable and productive partnerships between corporations and researchers in the behavioral sciences from the perspective of academic researchers or as perceived by academic researchers. Our respondents did not provide a clear distinction between active and passive types of interactions with companies while sharing their opinions and attitudes. Therefore, the analysis in this section will generally refer to both and make distinctions within the analysis whenever suitable.

In addition, data analysis did not reveal significant differences in attitudes between Swiss and American researchers. Despite the different continental affiliation of half of the sample from the other, scholars from both countries seem to have faced similar ethical reservations and technical challenges when considering academic-industry interactions. We hypothesized some of the reasons that might have contributed to this circumstance. First, the academic environment is an intrinsically international and dynamic reality, with researchers moving from one country to another and between the European and the American research scene. It was not uncommon for our participants to share that they had previously worked in different countries or were originally from a different continent. Secondly, the main ethical frameworks used in behavioral sciences are based on the Belmont Report (for the American side) and the Declaration of Helsinki (for the European side), which share numerous ethical principles and procedures (e.g., respect for persons/subjects, informed consent, and others).

Finally, the companies our participants explicitly mentioned were mostly American-based (Twitter, Facebook, Telegram, WhatsApp), which might have aligned opinions on corporate interactions. In addition, our sample, consisting of a limited number of participants also identified through snowballing, statistically could not identify a difference. As such, it would be of paramount importance to perform additional research that specifically investigates the attitudes of researchers from different countries towards cooperating with for-profit corporations. This will allow a better understanding of the different ethical and economic positioning towards corporate data to see what factors (country, discipline, ethical tradition) might influence them.

### The role of academic-corporate partnership and the value of using corporate data

Most participants considered both active collaboration with companies and passive use of corporate data as a promising, if not an essential, part of current academic behavioral research. Some of them emphasized the usefulness of the data that companies offer. Others highlighted the importance of industry resources towards developing truly impactful academic research on society, as companies have resources to invest in technologies needed for research, deliver the results of academic projects in the form of products (devices, algorithms, infrastructures), and collect and manage vast amounts of heterogeneous data. Specifically, in direct collaboration with companies, academic-corporate partnerships have been seen as an opportunity for academic institutions, companies, and society at large. A large corpus of studies has, in fact, proposed and critically analyzed models and strategies for sustainable and long-term collaboration between companies and academic research in medicine, chemistry, engineering, and biology. For instance, Bekelman, Li, and Gross (2003) conducted a systematic review analyzing the impact of financial conflicts of interest in biomedical research [15]. Dooley and Kirk (2007) proposed and analyzed the challenges and promises of a “triple helix” model of government-university-industry research collaboration [14]. Jain, Rosenblatt, and Duke (2014) analyzed the potential of Big Data and electronic health records to create new partnerships between university hospitals and pharmaceutical and device companies, by discussing the example of a five-year collaboration between the Indiana University School of Medicine and a global pharmaceutical company [13].

As seen in our results the drive towards collaboration has similarly polarized behavioral sciences such as psychology and sociology, with the advent of Big Data research. Such a shift has also been identified in a study by Davis and Binder (2016) on the rise of Corporate Partnership Programs (CPP) in university career centers. The study showed how companies in the US, traditionally more oriented toward technical universities and STEM programs, are starting to take an interest in academic institutions that include more liberal arts programs [17]. At the same time, in line with a study from Muscio and Pozzali (2013), academic researchers have highlighted some barriers to interaction with industry, such as finding appropriate business partners, the short-term orientation of industry research, different (on both sides) expectations and work priorities [54].

### Data quality and issues of reproducibility

Especially when it comes to the passive use of corporate data, where investigators are not actively involved in the procedures and methods for data collection, our study highlighted critical methodological challenges. Some of our participants raised the issue that data collected by companies is qualitatively not suitable for performing academically relevant scientific research. They highlighted that research practices in sociology and psychology are mostly theory- rather than data-driven and that the data provided by companies might lack essential information. The validity of knowledge based on big datasets and data-driven models is a discussion that has permeated the literature since the advent of the term Big Data. In line with our respondents, some studies claim that data offered by companies is biased and limited in its interpretability and that data-driven methods offer misleading results due to their tendency to mix up correlation and causation [1, 55]. Despite these concerns, the scientific community in the fields of sociology and psychology is finally recognizing the value of data-driven methods and new means of data gathering, such as access to corporate datasets, for research and is also exploring appropriate ways to merge more traditional theory-driven approaches with novel Big Data methodologies [4, 56].

Some researchers also related the issue of validation and reproducibility of research with the problem of access to corporate data. They were concerned that the difficulty or even impossibility of accessing corporate data, currently experienced by academic researchers, might compromise scientific validity. Schroeder (2016) has similarly argued that companies’ protectiveness about sharing data is problematic for the progress of scientific knowledge since it may be impossible to replicate studies or make their methods public [57]. This issue of corporate access to data and methods was also raised in the context of the Google Flu Trend (GFT), a study that aimed to provide real-time patterns of influenza activity. The study ultimately failed because of the dynamism of the algorithm used by Google, which was constantly changed and improved by the company. However, scholars argued that the lack of transparency of Google regarding their supporting materials and methods presented a barrier to replicability for researchers outside of the company that prevented the initial vision of GFT from being developed and perfected into a more accurate or even working model for flu prediction [58].

### Data sharing and conflict of interests

Our respondents saw the reticence in sharing corporate databases as an exclusion of the greater research community from valuable research data that might result in a disadvantage for scholarly investigation. Dooley and Kirk (2007) claim that one of the biggest drivers behind companies’ reluctance to share data with researchers is a consequence of conflicting interests/desires between the two actors. The industry wants to maintain secrecy to secure intellectual property rights and keep a competitive advantage. At the same time, academics aim to publish their results to validate their research and to advance both scientific knowledge and their academic careers [14]. Some of our participants voiced this conflict of interest and complained that companies were not too keen on providing data or they were not allowed to publish results stemming from company research.

We argue, however, that these different interests might be used to properly plan advantageous data-sharing strategies between academic institutions and private companies. For instance, as pointed out by one of our researchers when discussing the concept of the “research clock”, academia and companies work on two different timescales, with academic research generally “lagging behind” the companies’ schedule and interests [59]. This time gap could assist in sharing “old” data that is no longer considered sensitive by the company’s standards but is still valuable for academic research. At the same time, an interesting tension emerged from our results where some researchers claimed not to trust companies as they offer fewer protections to their users. In contrast, others, in the context of data sharing, argued that companies refrain from giving access to their data to protect their users’ privacy from further scrutiny. This concern is in line with a recent paper from Sikorska et al. (2020) that argues that reasons for reticence in data sharing include lack of trust, loss of privacy, especially risk of re-identification, and risks to regulatory compliance associated with how researchers use their data, in addition to the aforementioned inadequate economic incentives [60]. This tension only highlights the need to build a framework of trust and transparency to incentivize proper collaboration.

### Transparency in corporate research

Furthermore, the results point to an interesting tension: while many researchers voiced openness towards a possible active partnership with private corporations, they also expressed multifaceted ethical concerns and reservations linked to transparency of motives and research practices, consent, and anonymity. This should not come as a surprise, given that academic researchers are used to and trained to abide by a specific range of ethical standards that companies often do not need to consider. In line with our results, it is often argued that academic scholars are generally held to a higher ethical standard than industry researchers [28], while companies generally tend to fail to acknowledge the moral nuances behind for-profit corporate decisions. In a recent study on the morality of predictive models, Kiviat (2019) highlighted how corporations tend to protect themselves behind the claim of objectivity in algorithmic prediction just because it suits their for-profit motives, thus failing to consider that the mathematical objectivity of algorithms is at the core of many practices of unfair and unequal treatment [61, 62].

In the context of research ethics, transparency is often intended as a flexible principle that brings together different ethical components related both to the intent of research (what you are doing with the data and why) and practice (how you are getting the data–informed consent–and how you are processing it–data anonymity). This principle is currently considered a paramount component of research integrity by the academic online-data research community [28]. However, our respondents noted that transparency of motives and practices is generally not associated with corporate research. In his paper on the ethics of Big Data research, Rothstein (2015) shares this concern when he criticizes the practice of performing research behind the user’s back. This happened in the case of the Facebook Contagion Experiment or the OK Cupid website, where they publicly admitted to manipulating what was shown to their users to test and enhance their matching algorithms [18, 63]. Also, the risk of having corporate motives and incentives creeping into academic work and compromising research integrity was considered a significant hindrance to corporate collaboration. Unfortunately, several recent reports [15, 64, 65] highlight how financial ties pose a threat to scientific integrity, such as distortedly reporting pro-industry conclusions. These transparency issues might refrain academic researchers from engaging in collaborative efforts with private corporations.

Our participants also raised consent and privacy issues when dealing with academic-corporate collaborations and social media research. Consent is among the most challenging ethical concepts in the context of Big Data research for a twofold reason: on the one hand, Big Data methods are designed to reveal unforeseen connections, patterns, and information from the data, which makes it difficult for researchers to clearly delineate, at the time of consent, what will be the nature of the information that will emerge from a study and/or an experiment [55]; on the other hand dealing with consent in corporate data, poses challenges to consent practices since the subjects/users might be unaware of the details regarding how their data is being and analyzed and, most times, lack the appropriate control over their data [19, 66]. Closely connected to consent are issues of privacy in corporate Big Data research as studies could disclose private and sensitive information about the users/subjects, again due to the unpredictable information that will emerge from analysis [22, 67].

Despite these inherent challenges, a recent study by Hemphill, Schöpke-Gonzalez, and Panda (2022), which explored users’ feelings about social media data privacy and use, showed how users consider their social media data to be "moderately sensitive" and in need of protection. As such, they prefer that researchers clearly articulate the benefits and risks of a research project and explicitly seek consent before conducting a study [68].

As a detailed examination of these points is outside this manuscript’s scope, we refer to related literature that discusses both these two topics more in-depth [4, 29, 32, 69, 70]. For a more in-depth analysis of consent and privacy, we refer to our previous paper from this research project [46].

### Increased oversight for corporate research

Many respondents complained about being subject to more restrictive regulations than private firms and were concerned about the absence of regulatory oversight for corporate research. The lack of ethical evaluation for corporate research practices is becoming extremely problematic as private firms increasingly collect and analyze sensitive data from their users. On top of the risk of unethical studies, corporate research faces a growing societal backlash as scholars and the media are accusing companies of conducting unethical and harmful research [18, 71]. As such private–academic research partnerships might become a source of additional confusion within the already complex realm of regulatory practices in social computational and psychological Big Data research [22] and create reputational issues for academic scholars. The latter might inadvertently be involved in ethically opaque research or be accused of seeking partnerships with companies as a strategy to avoid research regulations.

For instance, this happened with the Facebook emotional contagion study. Although in line with regulatory standards, the study still raised ethical concerns within the academic community and society [38, 72, 73]. In that experiment, Facebook’s data collection practices were not fully consistent with research ethics principles such as informed consent [74]. Nevertheless, the Cornell University IRB did not flag the experiment as they "determined that the project did not fall under Cornell’s Human Research Protection Program" because Facebook conducted it for internal purposes. The Proceedings of the National Academy of Sciences of the United States of America (PNAS) therefore deemed it appropriate to publish the study. However, they admitted Facebook data practices to be a matter of concern [74].

However, a couple of respondents, hinted at the fact that the industry also has a reputation to protect. While academic research, as argued earlier, is usually considered more “ethical” [28], the private sector is setting up mechanisms to actively take responsibility to “respect, protect, and remedy human rights” [72]. Facebook, for instance, has set up an internal review process as a response to the public outcry that followed the emotional contagion experiment.

As the evidence of possible harm from corporate research is growing, increased regulatory measures for corporate research should be taken. Practical approaches to forming company review committees are currently being proposed to bring company practices into frameworks of trust and accountability [22, 75, 76]. For example, the institution of structures similar to IRBs within private corporations could benefit collaboration between companies and institutions as they could flag ethical/regulatory inconsistencies and issues promptly, facilitate the setting of common standards and goals, and provide a mutually shared regulatory and ethical framework [77]. Another important tool that has been used increasingly in the past years is external private independent IRBs such as Advarra Inc., and the Western Institutional Review Board (WIRB)–now known as WIRB-Copernicus Group (WCG IRB). Since these corporations aim to provide a thorough ethical review of research projects, they could be a way of enhancing collaboration and trust between academia and research when joining in a research project.

### Creating a sustainable space for academic-corporate interactions

The ethical and methodological tensions that emerge from this study raise the question of whether collaborations with corporations are really of value for the academic environment and what (if any) sustainable space can be created for both active and passive interactions between corporations and academia. According to Mittelstadt and Floridi (2016), a clear distinction should be drawn between “academic” and “commercial” research practices due to the different motivations that drive them: basic research to advance scientific knowledge in academia and product development and placement for profitmaking in the industry [55]. We do not believe that this distinction is practicable or even desirable. However, recognizing transparently and even exploiting this inherent difference could be considered a starting point to create sustainable, transparent, and ethical collaborations between companies and academia. This approach would allow us to have more realistic expectations regarding the different research approaches, aims, and goals between the two actors.

**Fig 1:**
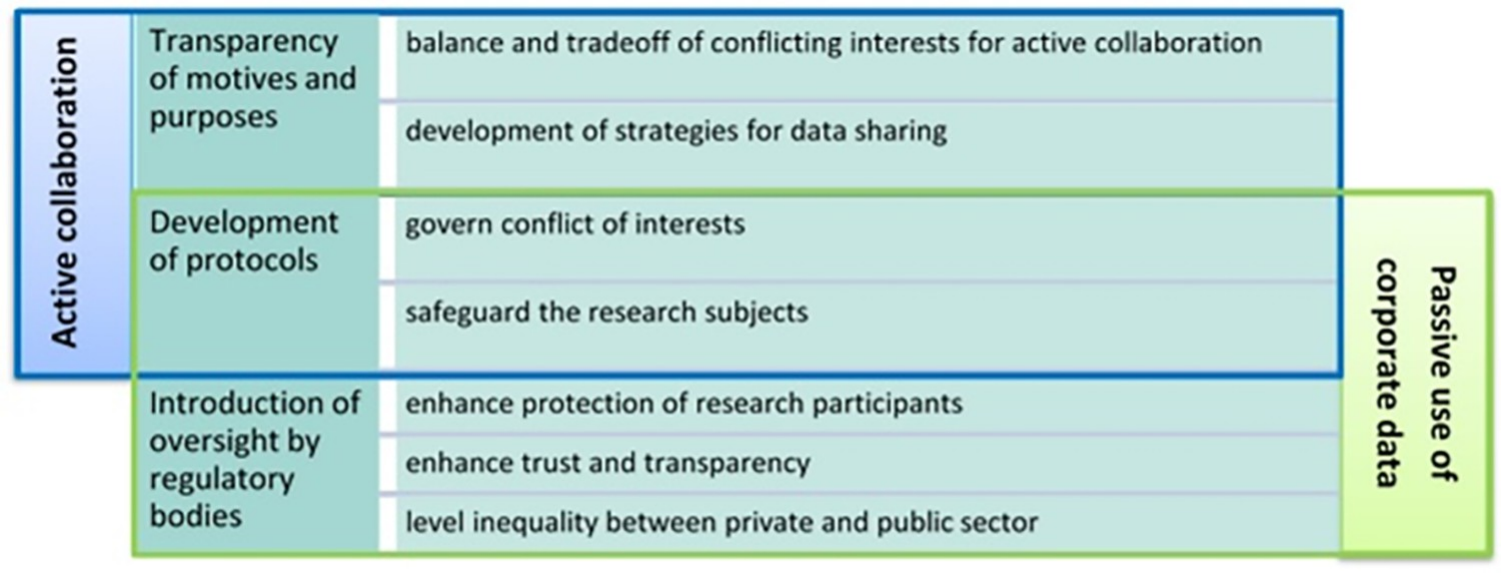
Suggestions to foster sustainable academic-corporate interaction.

The advent of Big Data especially has led to an overturning of the balance between applied and basic research by increasingly entwining industry and academic interests [78]. As such, a suitable space for interaction should be created. As one of our respondents noted, the time is ripe to ask critical questions about what data sources should be available for academic scholars, what type of collaborations scholars should be involved in, and what ethical framework should regulate academic-corporate partnerships. Based upon the discussion of our results, we provide a few suggestions on how to both improve active academic-industry collaboration and strategize dynamics for sustainable data sharing between corporations and academic institutions. Although far from being exhaustive, these suggestions represent a starting point to initiate a discussion on how to tackle this situation appropriately (Fig 1).

#### Transparency of motives and purposes

First, to enable sustainable active partnership, it becomes paramount to ensure transparency of motives, purposes, and interests when starting a collaboration between an academic institution and a company. Finding an appropriate balance of objectives and value systems between the two sectors is challenging. However, leading technology companies increasingly consider their commitment to the public good important [79, 80] and are more accepting of ethically sustainable collaborations. Mitroff and Sharpe (2017), for instance, provide an example of a successful partnership and give some suggestions to scholars on how to set up such a collaboration. These include choosing the right industry partners—usually the ones that have an established useful program for the research project that they are willing to share with academics—and aiming to achieve both theoretical and practical advances to satisfy industry expectations and interests as well [40]. In addition, strategies on how to sustainably share corporate data with academics should be explored further. For instance, the exploitation of the aforementioned “research clock” mechanism could be investigated to align some of the goals of companies and researchers (see Fig 1: “Active collaboration”).

#### Development of protocols

Secondly, appropriate protocols should be implemented to govern possible conflicts of interest, safeguard the human subject, and appropriately balance scholar’s ethical and legal concerns and the industry’s fear of overregulation. A study by Bekelman et al. (2003) highlights how finding the right balance between the two actors can sometimes be challenging to obtain. Academic researchers often consider proposed regulations ethically too loose, while the industry considers them too restrictive and an impediment to innovation [15]. The development of appropriate protocols thus becomes paramount both for active partnerships and passive use of corporate data. Without appropriate guidelines to regulate the former, the risk of having academic researchers undergoing undue influence from industry partners is high, especially when they depend upon companies for funding and essential infrastructures. When it comes to researchers accessing corporate databases, appropriate policies will provide academic researchers with the assurance that the data they are analyzing has been collected by following basic research ethics standards (see Fig 1: Overlap between “Active collaboration” and “Passive use of corporate data”). For instance, the DRAT (Data, Risk, Assessment, Tool) for university-industry collaborations developed by Sikorska et al. (2020) might prove to be an adequate step in this direction. This tool is set up to function as a medium to assess and control the risks associated with data sharing between universities and private companies, a task usually left to the individual corporate managers whose attitudes and motives for data sharing vary widely [60]. It would also be of paramount importance to investigate more closely the practices already put in place by private corporations to determine the appropriate standards to conduct research and initiate collaboration with private researchers.

#### Introduction of oversight by regulatory bodies

Third, in parallel with the development of policies, the implementation of review practices for corporate research would promote sustainable interaction and ethical research. As concerns of harm for research participants are emerging in corporate research, comprehensive oversight by regulatory bodies, either internal to the corporation [77] or instituted by third parties [75], should be put in place for the safeguarding of human subjects [71]. Both approaches, either having an external or an internal review committee, come with several complications that need to be addressed—for instance, issues of funding for the former and undue influence for the latter. However, the introduction of ethical review in corporate research would be valuable on many levels, especially for implementing data-sharing strategies between corporations and scholarly institutions and for viable academic use of corporate data. It would prove essential to predict and avoid the harm that could result for the users in specific data research practices; it would enhance transparency and trust between the different stakeholders involved in the research endeavor—academic partners, companies and their users/research subjects; it could assist in avoiding societal backlash, scandal and loss of reputation for both academic scholars and corporations; and it would level the current inequality of regulatory oversight between public and private entities (see Fig 1: “Passive use of corporate data”).

## Limitations

The first limitation of this study relates to the broad “umbrella” definition of Big Data utilized in this manuscript and in the overall research project. As mentioned in the methods section, we defined Big Data as “an overarching umbrella term that designates a set of advanced digital techniques (e.g., data mining, neural networks, deep learning, artificial intelligence, natural language processing, profiling, scoring systems) that are increasingly used in research to analyze large datasets with the aim of revealing patterns, trends, and associations about individuals, groups, and society in general” [47]. Especially in this manuscript, this broad definition did not allow for a nuanced analysis of the different types of data used by our participants and their specific characteristics and features—such as the different ethical challenges posed by high-risk data (financial and medical) versus minimal risk-data (social science and anonymized data). Future research on the topic will benefit from a more specific distinction and will provide additional insight into the specific challenges that emerge from different data types. In addition, our results are not generalizable to the opinions of the entire academic community due to several methodological choices, including the size of the interviewed sample, the focus limited to psychology and sociology as research fields, and the restriction of the recruitment to solely two countries, Switzerland and the United States. Therefore, future research should aim at providing a complete picture of how scholars perceive the opportunities and challenges of corporate partnership by expanding the investigation to other disciplines–such as computer science, biomedical informatics, physics, mathematics, and medicine–and other countries with different cultural and ethical backgrounds.

Secondly, some limitations emerge as a consequence of the overall aim of the project this manuscript stems from. The data used in this manuscript comes from a larger project designed to investigate the regulatory and ethical issues of Big Data (see details in the methods section). Therefore, the study was not designed to perform an in-depth exploration of scholars’ personal experiences with private firms nor to explicitly analyze the differences in attitudes between active and passive interactions with corporations. In addition, due again to the scope of the study, our sample did not exclusively include researchers involved in corporate collaboration, as the interviews we performed did not focus on this topic alone. Our sample included researchers who entered into collaboration with a private company and some who did not. We could not record as demographic data whether the participant was collaborating with a specific company due to the open-ended nature of our interviews, where participants were allowed to freely discuss topics pertaining to Big Data research, including personal experience with company collaboration and more general opinions regarding corporate practices. Consequently, our findings only mapped the opinions of academic researchers on academic-industry collaborations in general. Further research should focus on the experiences of researchers with private corporations more directly by closely analyzing their experiences and by clearly mapping the specific challenges and opportunities provided by both active and passive types of collaboration with private firms.

Finally, our sample only included academic researchers, thus omitting the input of researchers and people working in corporations and industries. For instance, our results did not allow us to make any remarks on the challenges faced by industry to engage with academia. Therefore, it would be essential that future research investigates the opinions and experiences of people in the industry sharing their data with universities to discuss the corporate side of the issues presented in this study and, at the same time, enhance appropriate practices of collaboration with academic institutions.

## Conclusion

This research illustrates some challenges, tensions, and opportunities associated with partnership and data sharing between companies and academia. Our results highlight how academic researchers were generally open to the use of corporate data for academic projects as they recognized the value that corporate datasets and resources could have for the advancement of scholarly research. However, they often associated partnerships with companies with several challenges. They reported restrictions towards access to corporate data that could result in issues of scientific validity and disadvantage for the academic research community. Participants also shared several ethical reservations, such as a lack of transparency of motives and practices of companies, issues of consent and anonymity, and possible loss of establishing the integrity of research caused by companies’ for-profit motives. Finally, our results highlight a perceived regulatory inequality between the private and the public sector, as many of our participants voiced their concerns regarding the lack of ethical oversight in corporate research.

As Big Data and digital technologies are creating new opportunities and incentives for academics to partner with private firms, strategies can be articulated and accepted to enhance and improve sustainable and ethical interaction, despite the ethical controversy and conflict of interests that academic-corporate partnerships might and have raised in some cases [13, 15]. According to Lutchen (2018), the last decade has brought a burst in the number of research deals between companies and universities, with both sides looking for more long-term, collaborative relationships [12]. This research only illustrated the advantages of corporate partnership as perceived by and for academic researchers. However, there are increasing incentives for corporations to undergo partnerships with academic institutions such as access to cutting—edge research and talent, a focus on basic research that companies lately are neglecting in favor of product development [12], observation of scientific development, and knowledge-transfer from academia to private companies [81]. Additional research should investigate the point of view of corporations and private firms to understand their opinions regarding academic-corporate collaborations and what appropriate strategies could be arranged to foster sustainable and mutually beneficial interactions between the two actors.

## Supporting information

S1 File Interview guide. Semi structured interview guide that illustrates the main questions and themes that the researchers asked to the participants.
(DOCX)
